# Optimisation of the Ethanol Fermentation Process Using Hydrothermal Pretreatment of Cellulose Waste—Effect of Fermentation Pattern and Strain

**DOI:** 10.3390/molecules29225266

**Published:** 2024-11-07

**Authors:** Jun Zhou, Pin Lv, Binsheng He, Jingjing Wu, Gao Wang, Hongzhi Ma, Yueyao Wang, Guiyun Chen

**Affiliations:** 1 School of Management, Changsha Medical University, Changsha 410219, China; 15304690053@163.com (J.Z.); hbscsmu@163.com (B.H.); wujingjing2401@163.com (J.W.); wanggaoedu@163.com (G.W.); 2 Department of Environmental Science and Engineering, University of Science and Technology Beijing, Beijing Key Laboratory of Resource-Oriented Treatment of Industrial Pollutants, Beijing 100083, China; lp990910@163.com (P.L.); wangueyao@163.com (Y.W.); 3 Xinjiang Key Laboratory of Clean Conversion and High Value Utilization of Biomass Resources, School of Resource and Environmental Science, Yili Normal University, Yining 835000, China

**Keywords:** tissue paper, hydrothermal pretreatment, fermenting strain, fermentation mode, ethanol

## Abstract

Suitable fermentation substrates and fermentation modes can effectively improve the fermentation ethanol yield. In this study, we optimised the hydrothermal pretreatment conditions by orthogonal optimisation using waste tissue paper as substrate. These conditions consisted of 50 min duration in a high-pressure reactor with pure water as solvent at a temperature of 160 °C. The biomass to water ratio was maintained at a constant level. The cellulose content of the pretreated TP was 81.19 ± 4.06%, which was an increase of 21.59% compared to the blank control. The 72 h reducing sugar yield of pretreated TP was 0.61 g sugar/g paper, which was 38.64% higher than that of untreated TP. Subsequently, the pretreated TP was fermented under optimal conditions. The mixed group of Saccharomyces cerevisiae and *Candida shehatae* (SC) showed a distributed saccharification fermentation pattern, with an ethanol yield of 28.11 g/L in 72 h. On the other hand, the single Saccharomyces cerevisiae (S) exhibited a homobloc saccharification fermentation pattern, with an ethanol yield of 35.15 g/L in 72 h.

## 1. Introduction

The depletion of fossil energy sources and their pollution of the environment presents new challenges to the world’s current energy mix. In response, the use of bioethanol as an alternative to fossil fuels has emerged as a promising option [1]. Bioethanol has been recognised as the predominant biomass-based renewable fuel due to its low greenhouse gas emissions and reduced reliance on fossil fuels [2]. In previous studies, bioethanol has typically been produced through fermentation using sugar-based feedstocks, such as edible feedstocks or starch biomass. While sugar-based feedstocks have been found to result in higher bioethanol yields, this approach raises concerns about competition with food crops and potential threats to the food supply [3]. The conversion from first-generation to second-generation bioethanol has occurred. Unlike first-generation bioethanol, second-generation bioethanol production employs a significant quantity of non-food feedstocks, like lignocellulosic wastes, which are in abundance and are not used for food or feed purposes, with only residual biomass available for conversion. This is in contrast to third-generation bioethanol, which is less cost-effective [3]. The cost of waste lignocellulosic biomass materials presents potential for widespread commercial application from a sustainability standpoint [4]. Cellulosic ethanol is typically created through an anaerobic fermentation procedure [5]. The anaerobic fermentation process comprises four primary stages: initial physical, chemical, or biological treatment to facilitate enzyme accessibility to cellulose and hemicellulose. Then, saccharification occurs by applying enzymatic or acid hydrolysis to convert cellulose into glucose. The pentose and hexose sugars produced are then fermented to produce ethanol; The fermentation broth is distilled to obtain anhydrous or fuel ethanol following purification [6].

Waste paper constitutes a major fraction of municipal solid waste, comprising over 35% of total lignocellulosic waste. Annually, more than 400 million tonnes of waste paper is generated worldwide, but only about 50–65% of this quantity is recycled [7]. Recycling waste paper has numerous benefits, although there are some limitations to consider. When pulp fibres are recycled beyond a certain amount, they become too short and keratinised for further use [8]. Unfortunately, this negative outcome often leads to waste paper being rejected as rubbish [9]. These waste papers and non-recyclables are transported to waste treatment facilities, such as landfills and waste incineration plants [10]. Nevertheless, landfill and incineration treatments are deemed environmentally unfriendly due to the production of polluting leachate and greenhouse gases [11]. Waste paper typically consists of 60–70% cellulose, with lower amounts of hemicellulose (10–20%) and lignin (5–10%) [12]. The simple composition of waste paper enables efficient resource utilisation, making it an environmentally advantageous option. However, most of the current research on the ethanol fermentation of waste paper is centred around office paper, and not much research has been done on the effect of different types of paper on ethanol fermentation, which is needed to determine the best substrate [13].

Waste paper is a potential feedstock for conversion to bioethanol due to its widespread availability, low cost, and high carbohydrate content. Additionally, it provides a practical and valuable alternative for managing surplus paper [14]. However, the structural conformation and crystalline nature of waste paper impede its effective value-added utilisation [15]. The complex structure formed by the interaction of its cellulose chains with hemicellulose and lignin causes low enzyme digestibility, making it challenging to hydrolyse it into fermentable sugars [16]. Appropriate pre-treatment methods are crucial for the successful hydrolysis of lignocellulosic biomass, as they can efficiently dissolve lignocellulosic components and disrupt the three-dimensional network of lignocellulose. Making cellulose and hemicellulose easily accessible to enzymes leads to a higher release of reducing sugars [17]. The resulting reducing sugar solution obtained during saccharification can be used as raw material for the production of liquid biofuels.

Appropriate pretreatment can effectively remove lignin fractions from biomass to promote hydrolysis and fermentation. Hydrothermal pretreatment is a cleaner form of physicochemical pretreatment. The process is conducted under high temperature and pressure with the objective of cleaving the glycosidic bonds between cellulose and hemicellulose in lignocellulosic biomass. Additionally, a portion of the lignin is degraded and removed, which can render the cellulose more receptive to cellulase activity. According to life cycle assessment analyses, hydrothermal pre-treatment is deemed as the most favourable discharge pre-treatment technology due to its ease of operation, short operating time, eco-friendliness, cost-effectiveness, and minimal equipment corrosion risk [18]. Hydrothermal heating has superior energy efficiency compared to conventional heating and is capable of improving cellulose hydrolysis and sterilising substrates. Nonetheless, the high energy consumption resulting from cellulose decomposition, which initiates at around 200 °C, must not be overlooked. At elevated temperatures, harmful substances such as HMF and phenolic compounds are formed, preventing the growth of microorganisms [19]. So, it is crucial to optimise the conditions of hydrothermal pretreatment for different substrates.

A complete fermentation process requires efficient fermentation strains in addition to substrates with high reducing sugar yields. During ethanol fermentation, yeast uses glucose to ferment under anaerobic conditions to produce ethanol and CO_2_. Microorganisms suitable for the fermentation of enzymatic hydrolysis products from paper should be able to ferment a wide range of sugars, since in addition to the main product glucose, xylose and cellobiose are also formed to a lesser extent by the hydrolysis of waste paper [20]. The growth and fermentation of Saccharomyces cerevisiae, which is currently used in industrial production, is also significantly inhibited by some of the by-products of hydrolysis, such as organic acids [21]. Therefore, constructing a strain or a mixed colony that can effectively ferment mixed sugars and resist by-product inhibition is important for industrial ethanol production. A mixture of *Pseudohyphae* and *Saccharomyces cerevisiae* can be used for ethanol fermentation using both glucose and xylose in the hydrolysate for higher ethanol yields.

In this study, orthogonal experiments were used to optimise the hydrothermal pretreatment conditions. Then, the use of a combination of Saccharomyces cerevisiae and Candida shehatae enables the utilisation of both glucose and xylose from the hydrolysate for ethanol fermentation to achieve increased ethanol yields, and the optimal fermentation pattern was determined by combining the changes in concentration of organic acids, which are fermentation by-products, in the hydrolysate.

1 Optimised hydrothermal pretreatment conditions by the orthogonal optimisation method.
2 The reducing sugar yield of pretreated pulp was 38.64% higher than that of untreated pulp.
3 A mixed group of *Saccharomyces cerevisiae* and *Candida albicans* (SC) showing a distributed saccharification fermentation pattern.

## 2. Materials and Methods

### 2.1. Materials

Waste tissue paper was collected from a university campus in Beijing. Several other chemicals, including 3,5-dinitrosalicylic acid, sodium hydroxide, potassium sodium tartrate tetrahydrate, phenol, anhydrous sodium sulphite, lactic acid, choline chloride, citric acid (CA), and sodium citrate, were used for the experiment. The chemicals were purchased from the supplier McLean and Aladdin Biochemicals Co. Ltd. located in Shanghai, China. In addition, cellulase (powder, 10,000 U/g, C805042-25g) and *β-glucosidase* (100 u/g, *Aspergillus niger*, S24786-100g), which is a commercial enzyme from Source Leaf brand, were used for the experiment. *Pseudohyphae* (*Candida shehatae*-*CICC 1766*) used in the experiments was purchased from the China Industrial Microbial Strain Preservation and Management Centre.

### 2.2. Compositional Analysis of the Material

The raw cellulose, hemicellulose, and lignin fractions and the pretreated cellulose, hemicellulose, and lignin fractions of tissue paper were analysed using the ANKOM220 Fibre Analyser using the Van Soest method. The dry matter of the materials used was converted to a constant weight at 105 °C by the standard method (ASTM D29741987). Ash content was determined according to standard methods (ASTM E17552007) and expressed as a percentage of residue remaining after dry oxidation at 550 °C. Equipping of ionic liquids and Deep Eutectic Solvent (DES) mixing with choline chloride (solid) and lactic acid (liquid) at a molar ratio of 1:2 were employed.

### 2.3. Chemical Composition Analysis

The severity factor (SF) is log Ro, a unique factor that couples temperature effects and reaction time, and it is used to review hydrothermal treatments, which helps in the optimisation of operating conditions, as well as comparative analyses of other hydrothermal pretreated biomasses and as an amplification parameter. So, the severity factor was defined as the main factor explaining hemicellulose dissolution in hydrothermal pretreatment.

The severity factor for hydrothermal pretreatment was calculated according to the following equation:

(1) $$\mathrm{SF}=\log_{10}R_{0}\;{=\log}_{10}(t\times \exp\frac{T-100}{14.75})$$

t: time, h; T: temperature, °C.

The expected monosaccharide products of hydrolysis were glucose and xylose and a few other sugars, all of which are reducing sugars. Therefore, for the determination of total hydrolysis products, the DNS Vis spectrophotometric method was used Ghose, 1987) [22]. Finally, the yield of reducing sugars in the pretreated TP was calculated from Equation (2) by the equation fitted to the reducing sugar standard curve. Q(g sugar/g paper) was used for detection.

(2) $$Q(g\;\mathrm{sugar}/g\;\mathrm{paper})=\frac{q\times d\times V}{v\times R}$$

Q: reducing sugar yield, g sugar/g paper; *q*: calculated amount of reducing sugar, mg; *d*: dilution factor; *V* is the volume of solution, L; *v*: volume of sample, mL; *R*: initial mass, g.

For the determination of glucose and xylose during yeast fermentation, a high-performance liquid chromatograph (HPLC) was used to monitor the glycation kinetics by applying high-performance liquid chromatography (HPLC-20AT, Shimadzo, Tokyo, Japan) with a refractive index detector (RID-10A, Shimadzu, Japan); the column was a Shodex SH1011 column (Showa Denko Co., Ltd., Tokyo, Japan). Three replicate experiments were performed for each enzymatic hydrolysis step. The concentration of ethanol was measured using a gas chromatograph (GC-2010plus, Shimadzu, Kyoto, Japan). Cellulose, hemicellulose, and lignin determination were performed using the ANKOM220 Fiber Analyzer with the Van Soest washing method for raw material fractions and pre-treated fractions.

### 2.4. Pretreatment of Waste Paper

The collected waste paper was put into the grinding machine (YMJ-001) to crush the waste paper to make the particle size uniform. Prior to hydrolysis, the material required pre-treatment to enhance the accessibility of cellulose to the cellulase complex. A total of 4 g of waste paper was mixed with 70 mL of the deionised water and then evenly distributed into a 100-millilitre autoclave reactor liner. The liner was placed into an autoclave reactor (CY-03628) and held at a temperature of 160 °C for 50 min. After cooling had occurred, the liquid and solid components were vacuum filtered with deionised water.

### 2.5. Enzymatic Hydrolyses

Enzymatic hydrolysis of untreated and pretreated waste paper was carried out by employing 4 g of waste paper (dry weight) in 100 mL jars at 8% (*w*/*v*) biomass loading. At 50 °C, 50 mL of citric acid–sodium citrate buffer was added to maintain a proper enzymatic environment (pH = 4.8). The jars were then placed in a heated magnetic stirrer (84-1A) at 100 rpm for 72 h. Waste paper was loaded with cellulase at 50 filter paper unit (FPU)/g and β-glucosidase at 25 FPU/g. Samples were extracted at 6, 12, 24, 48, and 72 h, respectively. When the desired enzymatic time was reached, the reaction was stopped by removing 0.1 mL of the supernatant and sealing it in a boiling water bath for 5 min. YM medium can be obtained.

### 2.6. Fermentation

In two 100 mL jars, 2.1 g of YM medium and 80 mL of deionised water were added, and they were autoclaved for 80 min; then, 0.5 mL of Candida shehatae sap and 0.05 g of dried Saccharomyces cerevisiae powder were added after sufficient cooling, and the culture was cultured for 28 h at 35 °C, 140 r/min, to reach the logarithmic growth period.

Four 100 mL jars vials with a working volume of 50 mL were filled with 50 U/g of cellulase and 25 U/g of β-glucosidase. Group A had 5 mL of Saccharomyces cerevisiae liquid (S) added to it, and it was subjected to Separation hydrolysis and fermentation (SHF) at a solids loading of 8% (4 g/50 mL). Group B had 2.5 mL of Saccharomyces cerevisiae liquid and 2.5 mL of *Candida shehatae* liquid mixture (SC) added to it, and it was subjected to SHF at a solids loading of 8% (4 g/50 mL). Group C had 5 mL of Saccharomyces cerevisiae liquid (S) added to it, and it was subjected to SSF at 8% (4 g/50 mL) solids loading. Group D had 2.5 mL of Saccharomyces cerevisiae liquid and 2.5 mL of *Candida shehatae* liquor mixture (SC) added to it, and it was subjected to SSF at 8% (4 g/50 mL) solids loading. The SHF was carried out at 50 °C, 80 r/min for 48 h followed by fermentation at 35 °C, 80 r/min for 24 h; the SSF was carried out at 35 °C, 80 r/min for 72 h.

### 2.7. Orthogonal Design

The pretreatment process of TP was optimised using the orthogonal test with hydrothermal solvent, hydrothermal temperature, and hydrothermal time; reducing sugar yield was selected as the index. The experimental protocol design was carried out using the L9(34) orthogonal test table to explore the best combination of factors in Table 1.

## 3. Results and Discussion

### 3.1. Basic Quantitative Compositional Analysis of the Material

Cellulose represents the glucose content, while hemicellulose represents the xylose content in the following enzymatic process. The reducing sugar solution produced in the process of saccharification of waste paper is the basis of developing liquid biofuel [23]. The analysis results of cellulose, hemicellulose, and lignin in waste paper towels after initial and pretreatment are shown in Table 2. The cellulose content of the initial paper towel was 59.60 ± 2.98%, and the lignin content was 29.93 ± 1.50%. After hydrothermal pretreatment, the cellulose content of TP was 81.19 ± 4.06%, which was increased by 21.59%. The lignin content was 11.73 ± 0.59%, which decreased by 18.2%. Hydrothermal reaction (HTP) was carried out under high temperature and pressure conditions, in which water in a solution breaks down into hydrogen ions and hydroxide ions. This reaction catalysed the hydrolysis of hemicellulose and lignin in lignocellulosic biomass, while removing the acetyl group, resulting in a significant increase in delignification efficiency and promoting the solubilisation of hemicellulose [24]. The existence of lignin was an important factor hindering the saccharification of biomass. Therefore, it was essential to enhance the solubilisation of lignin by pretreatment to release cellulose and hemicellulose from lignin. This will increase the effectiveness of cellulase against cellulose-based biomass. A similar study confirmed that the lignin content in pepper residue after hydrothermal pretreatment decreased from 25.3% to 11.8% [25]. Kong [26] found soluble lignin and several cellulose and hemicellulose degradation products in the hydrolyzed products of corn straw pretreatment.

### 3.2. Effect of Various Hydrothermal Conditions on the Enzymatic Properties of TP

This study assessed the efficacy of the hydrothermal technique for extracting reducing sugars from tissue paper. Optimal experimental parameters were determined through orthogonal tests, and the severity factor (SF) of the hydrothermal pretreatment conditions was calculated. The key parameters were the hydrothermal solvent, temperature, and duration.

A prosperous pretreatment method needs to generate extremely digestible solids, with the availability of lignocellulosic biomass being essential in establishing the effectiveness of transforming it into bioproducts [27]. The amount of reducing sugar obtained through enzymatic hydrolysis serves as a precise indicator of the effectiveness of pretreatment. This study assessed the efficacy of the hydrothermal technique for extracting reducing sugars from tissue paper. The optimum test parameters were determined by orthogonal method, and the severity factor (SF) of the hydrothermal pretreatment conditions was also calculated. The key determinants of the pretreatment process were the hydrothermal solvent, temperature, and duration.

**Figure 1:**
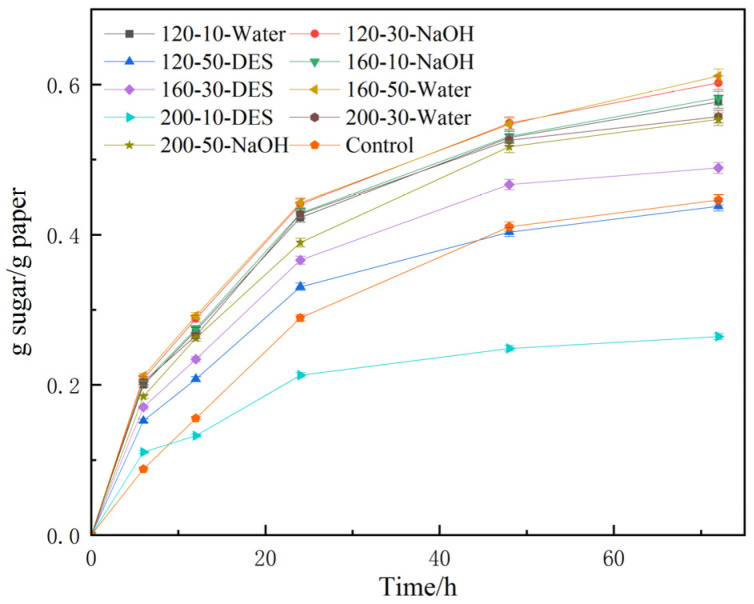
Orthogonal results of hydrothermal pretreatment conditions.

The samples for the orthogonal experiments were provided in the x-y-Z format. The results are depicted in Figure 1, where x denotes the temperature in degrees Celsius, y denotes the time in minutes, and Z denotes the hydrothermal solvent utilised. An instance of this representation is 120-10-Water, signifying the use of pure water as the solvent, a hydrothermal temperature of 120 °C, and a pretreatment time of 30 min.

From the analysis of Table 3, it can be inferred that the R-value determines the impact of the identified parameters on the results. A larger R-value implies a proportionally greater impact, whereas a smaller R-value represents lower impact. The greatest R value was observed for hydrothermal solvent at 0.185, succeeded by hydrothermal temperature at 0.103, and finally hydrothermal time at 0.075. The effect order on reducing sugar yield (g sugar/g paper) was C > A > B, meaning that hydrothermal solvent had the greatest impact on sugar yield reduction, followed by hydrothermal temperature, and finally hydrothermal time. Rahmani et al. demonstrated that the severity of temperature had a more significant impact during hydrothermal pretreatment in comparison to pretreatment time, given that pretreatment temperature holds a greater importance in lignin removal [28]. From the results obtained from optimising sugar yield, it can be inferred that the ideal combination for this study is A2B3C2. Combined with the data in Figure 1, this consists of a hydrothermal temperature of 160 °C, a hydrothermal time of 50 min, and pure water as the hydrothermal solvent. Moreover, the most significant yield of reducing sugar achieved under these conditions was 0.61 g sugar/g paper. Organic-acid-based DES disrupts the hydrogen bonding network between lignocellulosic components, while selectively solubilising specific components of the feedstock. However, it has the highest pKa of all tested solvents and ionises the largest amount of hydrogen ions to attack lignocellulose during pretreatment [29]. In addition, it should be noted that the presence of reactive reagents during ionic liquid pretreatment can have an inhibitory effect on hydrolases [30]. This explained that the enzymolysis results of the paper pretreated with hydrothermal-assisted organic acid–base DES were lower than those of the blank control group. The enzyme hydrolysis yield from the hydrothermal pretreatment with 1% NaOH as solvent was lower than that of the hydrothermal pretreatment with pure water as solvent due to the partial absence of hemicellulose in the TP. This is because the process by which NaOH breaks down lignocellulose involves breaking the α,β-aryl ether bond that links hemicellulose with lignin, thereby causing the separation and destruction of lignin, as well as the aldol substitution of hemicellulose [31]. Chen and colleagues achieved a glucose yield of 48.8% through enzymatic hydrolysis with hydrothermal pretreatment at 180 °C for 0.5 h [32]. Dimos et al. conducted diverse pre-treatments on corn stover. Out of all the pre-treatments, hydrothermal pre-treatment produced the most favourable outcomes in terms of ethanol production [33]. The findings indicate that higher sugar yields can be achieved for waste tissue paper through pre-treatment with pure water at appropriate temperatures without the addition of solvents or acidic and alkaline substances. This approach is deemed more economically and practically viable compared to the expense of other chemicals. Therefore, it is suggested that hydrothermal pretreatment presents an effective and environmentally friendly method for treating waste paper.

The ideal hydrothermal pretreatment condition for TP was established through one-way optimisation experiments, identifying pure water as solvent, and a temperature of 160 °C was maintained for 50 min. The resulting reducing sugar yield after 72 h was 0.61 g of sugar per paper, indicating a 38.64% increase when compared to TP that was untreated.

### 3.3. Fermentation of TP After Pretreatment

The products resulting from the hydrolysis of cellulose and hemicellulose are fermentable sugars, including hexoses and pentoses, that serve as optimal substrates for bioethanol production via fermentation. This process is anaerobic and can be carried out by various microorganisms [34]. The process of fermenting bioethanol is depicted in Equations (3) and (4) as follows:

(3) $$C_{6}H_{12}O_{6}\to 2C_{2}H_{5}OH+2CO_{2}$$

(4) $$3C_{5}H_{10}O_{5}\to 5C_{2}H_{5}OH+5CO_{2}$$

Simultaneous saccharification and fermentation (SSF) and separate hydrolysis and fermentation (SHF) are two classical processes used to convert pre-treated biomass to ethanol. In the SHF, the saccharification and fermentation processes will be conducted separately. This method offers the benefit of utilising two separate reactors for both the saccharification and fermentation processes, allowing them to take place under their respective optimal conditions. However, the elevated concentration of glucose, which would be generated while saccharifying cellulose, can impede enzyme activity and ultimately prohibit the ongoing saccharification of crystalline cellulose [35]. This problem can be overcome by a one-stage mixing process, often referred to as simultaneous saccharification and fermentation (SSF). SSF is a commonly employed and cost-effective approach that includes combining hydrolytic enzymes with ethanol fermentation microorganisms for simultaneous saccharification and fermentation. SSF not only circumvents the inhibitory effect of elevated sugar concentration within the fermentation broth on the enzyme but also streamlines the reaction process, diminishes the reaction period, and furthermore curtails energy consumption [36]. Therefore, concurrent saccharification and fermentation (SSF) for bioethanol production from TP can significantly enhance the yield of monosaccharides and consequently bioethanol.

#### 3.3.1. Fermentation of Saccharomyces Cerevisiae Yeast (S)

**Figure 2:**
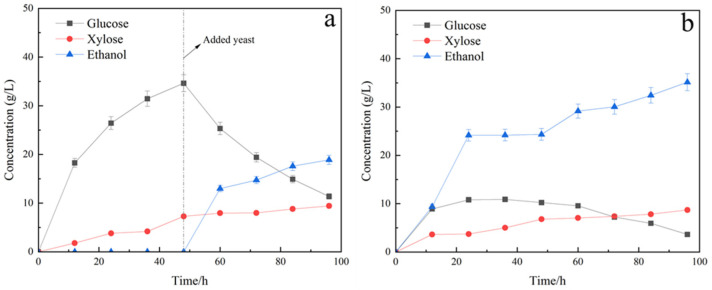
SHF and SSF fermentation of S: (**a**) SHF; (**b**) SSF.

In this section, we evaluated the feasibility of bioethanol production using pretreated TP as feedstock and Saccharomyces cerevisiae as the main functional group using SHF and SSF methods. The experimental results are shown in Figure 2.

The figures illustrated in Figure 2a,b display the outcomes of SHF and SSF. The ethanol yields yielded through SSF surpassed that of SHF at the same duration of operation—35.15 g/L compared to 17.58 g/L, respectively. The glucose and xylose concentrations in the synchronous saccharification fermentation group were kept low, thereby effectively reducing enzyme inhibition from high reducing sugar concentrations and enhancing enzyme hydrolysis. Moreover, the rate of ethanol production was maximum between 0 and 24 h in both SHF and SSF fermentation modes, and it began to decrease gradually with the gradual reduction of substrate and the accumulation of ethanol. Xylose content showed an increasing trend in both SHF and SSF modes, indicating that Saccharomyces cerevisiae could only utilise the glucose in the hydrolysate for ethanol production, but not the five-carbon sugars in the hydrolysate.

The optimal fermentation pattern for Saccharomyces cerevisiae was SSF with an ethanol yield of 35.15 g/L in 72 h. This converts to a substrate yield of 0.44 g ethanol/g substrate. Ethanol yields in both models exceeded those reported in prior studies. Lee et al. utilised the SSF method with oxalic acid pretreatment to generate an ethanol concentration of 20 g/L in 48 h. Pichia yeast-secreted extracellular β-glucosidase was observed to expedite and amplify ethanol fermentation beyond the anticipated theoretical yield attributable to its fibre disaccharide hydrolysis activity [37]. Sharma obtained a maximum ethanol concentration of 13.05 g/L by the SSF process conducted at 38 °C for 72 h [38]. The dilute acid pretreated lignocellulosic biomass with a low xylan to lignin ratio of 0.3 is reported to be critical in achieving a higher ethanol yield (14.35 g/L) with the SHF process utilising *Picrytis cinerea* CBS 6054 [39].

#### 3.3.2. Fermentation of Saccharomyces Cerevisiae and Candida Shehatae (SC)

**Figure 3:**
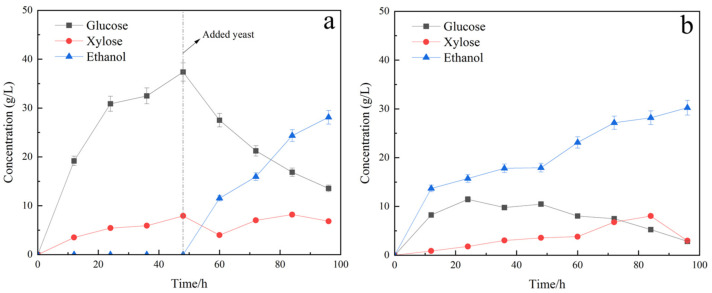
SHF and SSF fermentation of SC: (**a**) SHF; (**b**) SSF.

In this section, in order to make full use of reducing sugars in hydrolysate, we evaluated the feasibility of producing bioethanol by SHF and SSF using pretreated TP as raw material and SC as main strains. The experimental results are shown in Figure 3.

The findings of SHF for various strains are presented in Figure 2a and Figure 3a. In this mode, the ethanol production of mixed strains was 28.11 g/L, which is superior to that of single Saccharomyces cerevisiae at 17.58 g/L. The utilisation of xylose by Candida shehatae is the reason for this, as evidenced by the change in xylose profile shown in Figure 3a,b. After 48 h of saccharification, the glucose concentration of the single Saccharomyces cerevisiae group began to reduce as a result of fermentative utilisation by the yeast, whereas the xylose concentration continued to increase due to the continued enzymatic hydrolysis of hemicellulose. In contrast, the mixed group comprising Saccharomyces cerevisiae and Candida shehatae exhibited varying degrees of xylose concentration reduction, alongside an initial decrease in glucose concentration after 48 h of saccharification. Mixed-strain fermentation enables the speedy consumption of glucose and alleviates the inhibitory impact of glucose metabolism on xylose metabolism. This is because glucose inhibits the utilisation of xylose, and Saccharomyces cerevisiae can quickly use glucose and alleviate its inhibitory effect on xylose.

The outcomes of SSF of various strains are depicted in Figure 2b and Figure 3b. In the SSF approach, the ethanol yield of single Saccharomyces cerevisiae, at 35.15 g/L, exceeded the ethanol yield of mixed strains, which was 30.24 g/L. The inferior fermentation activity of Candida sheathes compared to Saccharomyces cerevisiae in the same environment is likely the cause, as demonstrated by the alterations in glucose and xylose profiles shown in Figure 3a,b. Although the group of single Saccharomyces cerevisiae was only capable of utilising glucose for fermentation, the concentration of glucose was kept low, signalling the high glucose utilisation ability of the Saccharomyces cerevisiae. The xylose concentration was sustained at a low level in the mixed group of Saccharomyces cerevisiae and Candida sheathe, with varying degrees of xylose concentration reduction observed. Although the presence of Candida sheathes utilised the xylose solution for fermentation, the mean value of glucose concentration was higher in the mixed group compared to the single Saccharomyces cerevisiae group, and according to Equations (3) and (4), the ethanol yield from 1 mol of glucose (2 mol) was higher than that from 1 mol of xylose (1.7 mol). So, it led to the phenomenon that the ethanol yield of the mixed bacteria group was not as good as that of the single brewer’s yeast group in SSF mode.

In both single and mixed groupings, the ethanol yields yielded through SSF surpassed that of SHF at the same duration of operation—35.15 g/L and 30.24 g/L compared to 17.58 g/L and 28.11 g/L, respectively. The glucose and xylose concentrations in the synchronous saccharification fermentation group were kept low, thereby effectively reducing enzyme inhibition from high reducing sugar concentrations and enhancing enzyme hydrolysis.

The optimal fermentation pattern for the mixed group (SC) of Saccharomyces cerevisiae and Candida shehatae was SHF with an ethanol yield of 28.11 g/L in 72 h; this converts to a substrate yield of 0.35 g ethanol/g substrate. The optimal fermentation pattern for single Saccharomyces cerevisiae was SSF with an ethanol yield of 35.15 g/L in 72 h; this converts to a substrate yield of 0.44 g ethanol/g substrate. Ethanol yields in both models exceeded those reported in prior studies. Lee et al. utilised the SSF method with oxalic acid pretreatment to generate an ethanol concentration of 20 g/L in 48 h. Pichia yeast-secreted extracellular *β-glucosidase* was observed to expedite and amplify ethanol fermentation beyond the anticipated theoretical yield attributable to its fibre disaccharide hydrolysis activity [37]. Sharma A et al. obtained a maximum ethanol concentration of 13.05 g/L by the SSF process conducted at 38 °C for 72 h [38]. The dilute acid pretreated lignocellulosic biomass with a low xylan to lignin ratio of 0.3 is reported to be critical in achieving a higher ethanol concentration (14.35 g/L) with the SHF process utilising *Picrytis cinerea* CBS 6054 [39].

### 3.4. Organic Acids During Fermentation

During ethanol fermentation, the main products are ethanol and carbon dioxide, accompanied by the production of other by-products. The process of ethanol fermentation in yeast cells produces by-products such as formic acid, acetic acid, propionic acid, lactic acid, glycerol, and heteroalcoholic oils [40] The organic acids in the hydrolysate were mainly derived from the incomplete tricarboxylic acid cycle metabolic pathway of yeast cells in the micro-oxygenated ethanol fermentation environment, and these toxic by-products accumulated during the fermentation process and may have certain inhibitory effects on yeast growth and ethanol fermentation. By analysing the concentration of organic acids in the hydrolysate, the activity of yeast cells can be indirectly reflected.

**Figure 4:**
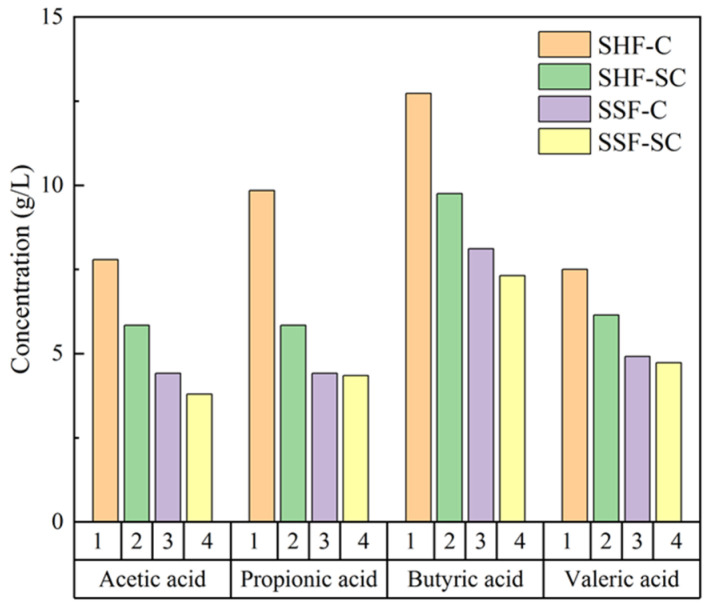
Average organic acid concentration in fermentation broths.

As analysed in Figure 4, lower concentrations of organic acids were present in the hydrolysate, with the highest concentration being butyric acid, which was produced mainly as a result of yeasts intervention due to open culture [41]. In contrast, organic acids such as acetic acid and propionic acid in solution are produced by the incomplete tricarboxylic acid cycle metabolic pathway of yeast cells. In terms of fermentation patterns, the SSF pattern produces less organic acids compared to the SHF pattern. This also corresponds to the SSF in Section 3.3, which produces more ethanol in the same amount of time. This is because the effect of organic acids on ethanol fermentation is due to an inhibitory effect on enzyme activities in the glycolytic pathway [42]. In terms of fermentation strains, mixed strains (SC) produced less organic acids compared to single brewer’s yeast (C), suggesting that mixed strains play an important role in reducing by-product inhibition and are more conducive to lifting inhibition to promote ethanol yield. The concentration of by-products in the hydrolysate varies depending on the type of biomass and pretreatment process, most of which are acetic acid [43], where acetic acid is produced from acetyl xylan in hemicellulose [44]. Several studies have reported that genes metabolised by Saccharomyces cerevisiae respond more significantly to acetic acid during glucose fermentation [45]. When yeast fermented different sugars, i.e., a mixture of glucose and xylose, the mixed strain intensified the utilisation of xylose, reduced the conversion pathway of hemicellulose to acetic acid, and effectively lifted the inhibitory effect of organic-acid-based by-products on ethanol fermentation [46]. The results of Pozdniakova et al.’s analysis of the fatty acid synthase (FASI) of Saccharomyces cerevisiae also showed that yeast produces only a small fraction of fatty acids during fermentation [47].

The inhibitory effect of by-products, especially organic acids, on yeast can be achieved by changing the fermentation mode and strain, where the best fermentation mode is SSF, and the best fermenting strain is mixed strain (SC). Similar results were obtained with ethanol yield.

## 4. Conclusions

The hydrothermal pretreatment of waste paper towels was conducted under optimal conditions, resulting in a cellulose content of 81.19 ± 4.06%, an increase of 21.59% compared to the untreated sample, while the lignin content decreased by 18.2% to 11.73 ± 0.59%. This pretreatment not only sterilised and disinfected the paper towels but also enhanced enzyme accessibility. Orthogonal experiments identified the optimal conditions as 160 °C for 50 min using pure water as the solvent. The pretreatment yielded 0.61 g of reducing sugars per gram of paper after 72 h, representing a 99.07% conversion rate and a 38.64% increase over untreated samples. The bioethanol production methods employed demonstrated favourable results, with a mixed fermentation approach of Saccharomyces cerevisiae and Candida sheathe yielding 28.11 g/L ethanol in 72 h (0.35 g ethanol/g substrate). In contrast, the single strain fermentation of Saccharomyces cerevisiae achieved 35.15 g/L in the same timeframe (0.44 g ethanol/g substrate).

## Figures and Tables

**Table 1:** Orthogonal experimental factors and levels.

Level	Hydrothermal Temperature (A)	Hydrothermal Time (B)	Hydrothermal Solvent (C)
1	120 °C	10 min	water
2	160 °C	30 min	NaOH
3	200 °C	50 min	DES

**Table 2:** Quantitative compositional analysis of tested types of the material.

Material	Cellulose (%)	Hemicellulose (%)	Lignin (%)	Ash (%)
Tissue paper before treatment	59.60 ± 2.98	8.82 ± 0.44	29.93 ± 1.50	0.37 ± 0.019
Tissue paper after treatment	81.19 ± 4.06	5.74 ± 0.29	11.73 ± 0.59	0.59 ± 0.030

**Table 3:** The results of the orthogonal experiment of tissue paper for hydrothermal pretreatment.

Group	Hydrothermal Temperature (A)	Hydrothermal Time (B)	Hydrothermal Solvent (C)	Reducing Sugar Yield (g Sugar/g Paper)
1	1	1	1	0.577
2	1	2	2	0.602
3	1	3	3	0.438
4	2	1	3	0.581
5	2	2	1	0.489
6	2	3	2	0.611
7	3	1	2	0.264
8	3	2	3	0.557
9	3	3	1	0.553
k_1_	0.539	0.474	0.582	
k_2_	0.561	0.549	0.579	
k_3_	0.458	0.534	0.397	
R	0.103	0.075	0.185	
correlation	C > A > B			
Optimal level	A_2_B_3_C_2_			

## Data Availability

All data are fully available without restriction.
